# Organ donation in the time of COVID-19: the Israeli experience one year into the pandemic—ethical and policy implications

**DOI:** 10.1186/s13584-022-00519-8

**Published:** 2022-01-31

**Authors:** Eyal Katvan, Jonathan Cohen, Tamar Ashkenazi

**Affiliations:** 1 Bar Ilan University, Ramat Gan, Israel; 2 Peres Academic Center, Rehovot, Israel; 3 Israel National Transplantation Center, 15 Moses Street, Tel Aviv, Israel

**Keywords:** Organ donation, Covid-19 pandemic, Living kidney donation

## Abstract

**Purpose:**

To present the response of the Israel National Transplantation Center (NTC) to the evolving challenge of COVID-19, the impact on deceased organ donation and living organ kidney donation during 2020, and resultant policy and ethical implications.

**Methods:**

Data collected included (i) for deceased donors, the total number of potential organ donors, if hospitalized in ICU or general ward, cause of death, number of family authorizations and refusals, number of actual donors, number of organs transplanted/donor and total number of transplants performed; (ii) for living-kidney-donors (related or altruistic), the number of procedures performed; and (iii) the number of patients registered on the national organ waiting-list.

**Results:**

Following the first case (February 2020), deceased organ donation continued uninterrupted. The total number of potential donors was similar to 2019 (181 vs. 189). However, the number of families approached for donation decreased significantly (P = 0.02). This may be attributed to COVID-19-imposed limitations including fewer brain death determinations due to limited possibilities for face-to-face donor coordinator-donor family interactions providing emotional support and visual explanations of the medical situation. Fewer donors were admitted to ICU (P = 0.1) and the number of organs retrieved/donor decreased (3.8/donor to 3.4/donor). The overall result was a decrease of 24.2% in the number of transplant procedures (306 vs. 232). Living kidney donation, initially halted, resumed in May and the total number of procedures increased compared to 2019 due to a significant increase in altruistic donations (P < 0.0001), while the number of related-living donations decreased.

**Conclusion:**

This study of organ donation during a crisis has informed the introduction of policy changes in the NTC including the necessity to mobilize rapidly a “war room”, the use of innovative virtual tools for contact-less communication, and the importance of cooperation with hospital authorities in allocating scarce health-care resources. Finally, the pandemic highlighted and intensified ethical considerations, such as under what circumstances living kidney donation be continued in the face of uncertainty, and what information to provide to altruistic donors regarding a prospective recipient, in particular whether all options for related living donation have been exhausted. These should be addressed now.

## Introduction

Coronavirus disease 2019 (COVID-19) is a contagious illness caused by the severe acute respiratory syndrome coronavirus 2 (SARS-CoV-2). Since first identified in Wuhan, China, in December 2019, the disease has spread throughout the world, resulting in a still-ongoing pandemic with severe social, economic and medical consequences. Initial data from China found that 19% of infected patients required hospitalization while experience from the most severely affected countries, such as Italy, revealed that 3.2% of their patients required admission to intensive care units (ICU) to manage the respiratory consequences of the COVID-19 [1, 2]. Thus, it became apparent that the availability of hospital beds, in particular ICU beds, would become crucial to treat effectively those most severely affected by the virus.

Both potential organ donors and organ recipients are routinely managed in an ICU. In this regard, the Transplantation Society suggested temporarily suspending deceased organ donation programs in countries with widespread virus transmission and where resources for managing these patients may be limited [3]. Despite this, the steering committee of the National Transplantation Center (NTC) in Israel reached a unanimous decision at the onset of the outbreak in Israel, the first case being confirmed on 21 February 2020, that the organ donation program should continue without interruption, since organ donation is considered a life-saving procedure for many patients with end-organ disease. However, it was recognized that there were significant concerns relating to this decision which needed to be addressed. These related in particular to protecting both recipients from acquiring the disease via transmission of the virus from infected donors and health care workers involved in the donation process.

In this article we describe the changes in NTC policies during the crisis, the actions which the Center implemented in order to optimize results in a reality of rapid changes and the effect of the pandemic on organ donation in Israel. In addition, we identify issues which will need to be addressed in order to provide an appropriate response for future similar scenarios.

## Methods

### Testing for the Covid-19 virus

This was performed using the SARS-CoV-2 real-time reverse transcriptase polymerase chain reaction (PCR) obtained from a nasopharyngeal swab. All potential lung donors were required to have an additional negative test from a bronchoalveolar lavage-derived specimen. Regarding the availability of test kits, priority was always given to potential donors so that there was no instance where a donor was not tested due to unavailability of the test. Tests were performed within 24 h of the planned organ retrieval and results were available within 6–8 h during the first 4 months of the pandemic and since then within half an hour.

### Approach to donation from BD/NDD donors

At the beginning of March 2020, an ad hoc committee of the NTC was called into urgent session in order to produce guidelines relating to organ donation in the face of the pandemic. Participants included senior members of the Center, infectious disease specialists, the medical advisor to the Center and representatives of transplant surgeons and physicians. The guidelines were adopted forthwith and distributed to all medical personnel involved in organ donation and transplantation. Donation was disqualified from potential donors who (i) had a current proven infection with COVID-2019 as detected by PCR obtained from a nasopharyngeal swab; (ii) had been directly exposed to a person with a proven infection within the previous 14 days despite themselves having a negative test; (iii) had returned from travel abroad within the preceding 14 days, even in the absence of any symptoms and/or a negative test; and iv) presented with pneumonia or lower respiratory tract infection without a proven microbiological diagnosis. Donation was permitted from (i) donors with a pneumonia or lower respiratory tract infection where a microbiological diagnosis was made and in the presence of a negative test; and (ii) donors who had previously tested positive, after a period of 6 weeks had elapsed and 2 repeat tests were shown to be negative within 72 h of donation. All potential lung donors were required to have an additional negative test from a specimen derived from bronchoalveolar lavage. In addition, all potential donors in Israel routinely undergo a total body CT scan prior to organ retrieval. At the present time, Israel does not have an active DCD (donation after cardiac death) program.

### Approach to donation from living donors

Organ donation from living donors was halted at the beginning of March 2020 as were the national and international kidney pair donation programs. Following a period of lockdown during March, a significant decrease in the number of new cases diagnosed with COVID-19 was noted and living donation was resumed at the end of May 2020 following the adoption of new guidelines. Donation was permitted from (i) donors previously proven positive for COVID-19 and donors who had been exposed to a positive person provided that a period of at least 6 weeks had passed since the initial positive test, at which time two nasopharyngeal swab tests were shown to be negative and 2 further tests for the virus 1 week before the planned donation, with an interval of 24-hours between tests, were also negative; (ii) donors not residing in an endemic area and who had not come into contact with a confirmed COVID-19 positive person. In this last scenario, the donor was still required to have two negative tests with an interval of 24 hours between them (preferably as close as possible to the day of surgery). Donation was not permitted from donors who tested positive for the virus or who resided in endemic areas.

### The in-hospital donation process

In Israel, donor coordinators (DCs), who are who are trained and overseen by the National Transplantation Center, are responsible for all aspects of the donation process, including donor identification, medical management and optimization, the family approach and coordinating organ retrieval. The DCs typically accompany donor families from the time of donor identification to organ retrieval providing both emotional and logistic support. At the start of the pandemic, guidelines were issued to the DCs which included the compulsory wearing of full personal protective apparel at all times, COVID-19 testing once weekly and/or where exposure to a positive patient had occurred and/or in the presence of any symptoms of the disease, and limiting their on-site presence to < 8 h per day. At the outset of the pandemic, all major hospitals issued guidelines regarding hospital visitor restrictions whereby only one family member is allowed to accompany and visit an in-patient, and then only at certain times and only for a short duration. In addition, all patients and family members entering a hospital are required to undergo testing for COVID-19.

Every effort was made to admit all potential donors to an ICU. COVID-19 positive and negative patients were treated in separate ICUs. Where this was not possible due to a shortage of beds, donors were admitted to a general medical ward in which there were no COVID-19 positive patients. All heart, liver and lung recipients are routinely admitted to an ICU, whereas kidney recipients are only admitted if significant intra-operative complications occur. Most elective procedures in hospitals throughout Israel continued largely uninterrupted.

### Data collection

The following data was collected: (i) the number of the general population tested positive for COVID-19 per month and the number who passed away; (ii) for BD/NDD donors, including the total number of potential donors (a patient who meets the criteria for brain death with no absolute contraindications to organ donation) identified per month; mean age; number of potential donors found to be COVID-19 positive; where hospitalized, i.e. in ICU or general medical ward; cause of death (defined as due to trauma, anoxic brain damage or CVA [including ischemia and intracerebral bleeding]; number of family authorizations and refusals; number of actual donors (donor from whom organ/s retrieved and transplanted); number of organs transplanted per donor; organ utilization (total number of organs retrieved and transplanted/total number of donors × 100); total number of transplants performed; iii) for living kidney donors, including whether related or altruistic; the number of transplantation procedures performed; and iv) the number of patients registered on the national waiting list for all organs. All these data were compared to the corresponding period in 2019. Data was derived from the records of the NTC, the Ministry of Health, and the Central Bureau of Statistics.

### Statistical analysis

Results are expressed as the mean ± SD. Differences between the two time periods were calculated with the Chi-squared test for the significance of the difference between two independent proportions and the difference between the observed means in two independent samples, where appropriate. The significance level was set at < 0.05. Data were analyzed using SPSS 25 (IBM, US).

## Results

**Fig. 1:**
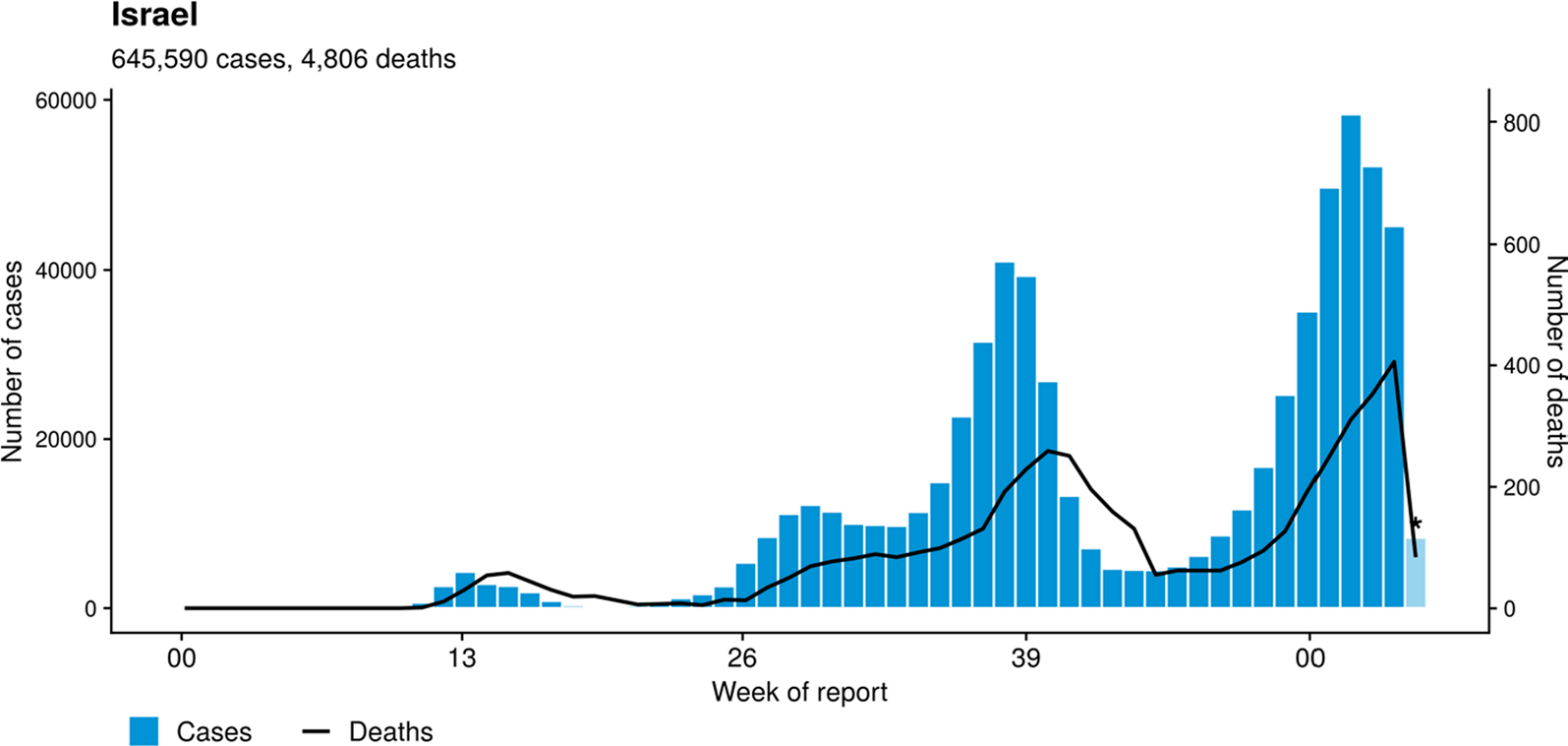
Number of new cases of COVID-19 and death rate per week, January 2020 to January 2021

Three major outbreaks occurred, the first in March 2020, the second in September 2020 and the third in December 2020, the last 2 of which were associated with significant increases in both the number of positive cases and the death rate (Fig. 1). These outbreaks were followed by general closures/lockdowns [4].

**Fig. 2:**
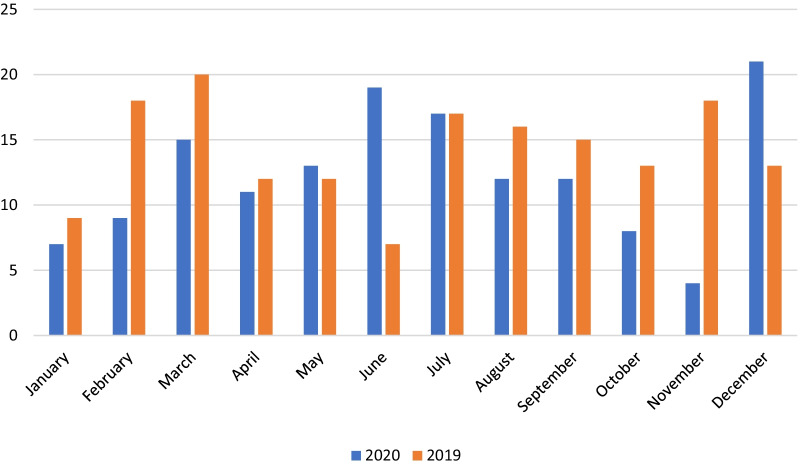
Number of potential organ donors by month, 2020 versus 2019

The number of potential donors according to month, 2020 versus 2019 is shown in Fig. 2. Two peaks in the number of potential donors in 2020 were noted, namely in June (46% more than in 2019) and December (24% more than in 2019).

Organ donation from BD/NDD donors continued uninterrupted throughout 2020 and whoever received an allocation of a deceased organ was transplanted. The data for the donation process in 2020 and 2019 are shown in Table 1. The total number of potential donors detected and the causes of BD/NDD were not significantly different in the 2 periods. Four identified potential donors were rejected as they were found to be COVID-19 positive. The number of patients where no family approach for organ donation was made increased from 17 (9.0% of the total potential donors) in 2019 to 29 cases (16% of the total potential donors) in 2020 (*P* = 0.04). There was a trend for less potential donors to be admitted and managed in an ICU in 2020 (90% vs. 95% in 2019, *P* = 0.10). The consent rate was unchanged over the 2 periods (58.7% in 2020 vs. 58.2% in 2019, *P* = 0.92). The mean actual donor age in 2020 was 53.8 ± 75.3 years compared to 49.8 ± 26.4 years in 2019 (*P* = 0.62). The number of organs retrieved and transplanted per donor decreased in 2020 by 10.6% compared to 2019 (3.4/donor in 2020 vs. 3.8/donor in 2019). There was a decrease in utilization of all organs from 2019 vs. 2020, which was significant only for kidney utilization (82% in 2019 to 71.8% in 2020; *P* = 0.03). The total number of transplants decreased by 24.2% from 306 in 2019 to 232 in 2020. No case of COVID-19 was reported in any recipient.

**Table 1:** Characteristics of organ donation, 2020 versus 2019

Parameter	2020	2019	*P* value
Total potential donors identified, n	181	189	
*Cause of BD/NDD (%)*			
CVA	44.2	40.2	0.44
Anoxia	31.4	33.8	0.62
Trauma	17.6	20.6	0.46
Other	6.6	5.4	0.63
Inability to perform apnea test, n	4	2	
No family approach, n (%)	29 (16%)	17 (9.0%)	0.04
Family approached, n (%)	148 (81.7%)	170 (89.9%)	0.02
Managed in ICU, n (%)	134 (90%)	161 (95%)	0.10
Consent, n (%)	87 (58.7)	99 (58.2)	0.93
Age actual donors, mean ± SD	53.79 ± 25.3	49.80 ± 26.4	0.62
No. organs transplanted per donor	3.4	3.8	
*Organ utilization (%)*			
Kidney	71.8	82	0.03
Lung	38.5	45	0.24
Liver	88	88	1.0
Heart	21.6	23	0.76
No. transplants performed	232	306	

*BD/NDD* brain death/neurologically declared death, *CVA* cerebrovascular accident

The results for living organ donation are shown in Table 2. There was a significant increase in the total number of procedures performed in 2020 (*P* < 0.0001). This was due to a significant increase in donations from unrelated altruistic donors (*P* < 0.0001).

**Table 2:** Number of living donations performed, 2020 versus 2019

Parameter	2020	2019	*P* value
Total	273	248	
Related, n (%)	85 (31.1%)	121 (48.7%)	< 0.0001
Unrelated, n (%)	188 (68.8%)	127 (51.2%)	< 0.0001

There was a 10% increase in the number of those waiting for a transplant, from 1153 in 2019 to 1266 in 2020 (Table 3). The number increased for every organ. The number of new patients who joined the national waiting list was similar in the 2 years (736 in 2020 vs. 737 in 2019). 63 patients died on the waiting list in 2020 (4.9%) compared to 50 in 2019 (4.3%) (*P* = .83).

**Table 3:** National transplant waiting list, 2020 versus 2019

Year	Total	Kidney	Liver	Heart	Lung	Heart/lung	Kidney/pancreas
January 2020	1153	857	86	71	124	7	8
January 2021	1266	917	110	93	131	6	9

## Discussion

Deceased organ donation in Israel continued uninterrupted during the first year of the COVID-19 pandemic. While there was no change in the total number of potential donors detected compared to a similar period in 2019, the number of actual donors decreased significantly, the result of a significant decrease in the number of donor families approached for organ donation. This, together with a decrease in the number of organs retrieved and transplanted per donor, resulted in fewer transplantation procedures being performed. Interestingly, the number of living kidney donations, in particular from altruistic non-related donors, increased significantly.

As the pandemic spread, many countries reported significant reductions in the number of deceased organ donors. Thus, for example, the mean number of deceased donors declined from 7.2 to 1.2 per day in Spain, while in France, a 16% decrease was noted [5, 6]. Possible explanations put forward for these changes included a decrease in the number of available ICU beds for non-COVID-19 positive patients, a decrease in the number of trauma patients (in particular road traffic accidents) and changes in acceptance criteria [6, 7]. In Israel, no change in the absolute number of identified potential donors was noted. Separate ICUs and general medical wards for COVID-19 positive and negative patients were established early in the course of the pandemic thus allowing the continued admission of non-affected patients, including potential donors. In addition, no decrease in the number of BD/NDD donors due to trauma was noted.

Despite these measures, compared to a similar period in 2019, the number of transplant procedures performed from deceased organ donors decreased by 24.8% from 306 to 232. This may be the result of several factors. Firstly, there was a significant increase in the number of cases where no approach was made to a donor family for donation, which was related in all cases to their non-acceptance of BD/NDD [8]. In this regard, studies have suggested that understanding the significance of BD/NDD may influence family decision-making regarding organ donation ^9^. However, restrictions imposed on both DCs and donor families in Israel resulted in limited opportunities for the DCs to offer detailed explanations regarding the potential donor’s status, in particular the significance of absent brain function. In addition, the DCs reported that the need to communicate telephonically with family members not in the hospital limited their ability to assess inter-family dynamics in the presence of refusal and to use aids such as CT scans to reinforce their message of severe brain damage. When it becomes clear that the family do not accept the diagnosis of BD/NDD DCs in Israel are instructed by the NTC not to make a family approach. The impact of limited DC-donor family contact was also noted in a recent web survey of 19 OPOs in the US [9]. In this study, initial family contact was made via telephone for 18% of potential donors and this, together with limited onsite interaction, was suggested as negatively influencing the donation process as evident by an 11% decrease in authorizations. It is clear that innovative technological solutions will be required to optimize virtual interactions with family members. Secondly, the number of organs retrieved and transplanted per donor n Israel decreased by 11% (3.4/donor in 2020 vs. 3.8/donor in 2019), which was especially pronounced for kidney utilization. This may be partly explained by the fact that while all potential donors were admitted to hospital, there was a trend for more to be managed in general medical wards (*p* = 0.1) where the intensity of care (both regarding nurse-patient ratio and possibilities of invasive monitoring) is much less than that of an ICU. As a consequence of the decreased number of transplants performed, both the number of patients on the national waiting list as well as the number who died while awaiting a transplant increased.

Regarding living kidney donation, this was initially suspended following the initial pandemic outbreak in March 2020 but resumed in May after a period of lockdown and a significant decrease in new COVID-19 cases. The program continued uninterrupted since that time and in fact, a significant increase was noted in the number of transplants compared to 2019, due to increased altruistic donations. Regarding the latter, their number has been steadily increasing over time, largely the result of the Gift of Life organization, started in 2011 by Rabbi Haber, himself a kidney recipient. The organization encourages altruistic kidney donation, initially amongst the Rabbi’s constituents, namely the ultra-Orthodox Jewish community, but latterly amongst the general Israeli public. The Rabbi passed away in April 2020 due to complications of the COVID-19 virus. In the weeks following his death, 100 additional altruistic donors registered with the NTC, some of whom were rejected on medical grounds while others successfully donated a kidney. A survey of DCs involved with living donation revealed that half ascribed the increase in donations to an act of solidarity with the Rabbi’s passing while a further half ascribed this to the opportunity provided by COVID-19 related work and study institution closures to donate without interruption of work and study schedules.

Since December 2020, a program of intensive vaccination against the virus has been successfully implemented in Israel (to date half the population have been vaccinated) and all potential recipients on the waiting list are being advised to undergo vaccination. Many questions remain to be answered, including whether vaccinated donors could be considered safe (both living and deceased) and guidelines are in the process of being drafted. Regarding living donation, it has been suggested that surgery be performed one week after both donor and recipient have received the second dose of the vaccine. In addition, while family members were till the present not permitted to enter the transplant wards, they will now be entitled to accompany the donor provided they have received 2 vaccinations and a repeat COVID-19 test is negative.

### Policy and ethical implications

#### Functioning in times of uncertainty

##### Ensuring a timely response

In conditions of a changing reality and uncertainty, as was experienced in the COVID-19 epidemic, dynamic and fast decision-making is essential. In this regard importance will be placed on the ability to convene relevant experts for a particular problem from all corners of the country at short notice. During the pandemic, the NTC was activated in the format of a “war room”, using virtual tools where necessary, and this proved to be effective in facilitating a rapid and appropriate response to the crisis. The Center will maintain and utilize this “war room” approach during routine as well crisis situations in the future so as to promote rapid decision-making and process implementation.

##### Extra caution—living donation

At the same time, decisions must be based on ethical grounds, to achieve a balance between uncertainty and the needs of patients and donors. This is particularly relevant for living kidney donation where healthy people who put themselves at potential risk in a state of uncertainty [10–13]. In such circumstances, the NTC decided to halt this activity until such time that decisions could be based on more updated acquired knowledge and then to formulate policies based on scientific facts. This same policy will be adopted in the event of similar future crises.

#### Scarce health resources

The crisis brought to light that even if there are organs for transplantation, they cannot always be utilized, since resources (including appropriate personnel, equipment and hospital ICU beds) may need to be allocated to unexpected crises at the expense of organ donation. Preparing ahead for such events will require close cooperation with senior hospital administration.

#### Preparing for conditions where relatives cannot or fear to be part of the donation process.

##### Donation from brain dead donors

COVID-19-imposed limitations resulting in fewer BD/NDD determinations due to limited possibilities for face-to-face DC-donor family interactions providing emotional support and visual explanations of the medical situation. In this regard, attention will be given to the development of innovative virtual tools for contact-less communication with deceased donor families.

##### Living donation

As noted, during the coronavirus crisis, a gap was created between the number of altruistic donors and related living donors. The latter may refrain from donating, for growing health concerns during this period when there is already one "patient" in the family who needs to be cared for. There may also be a fear among family members of a patient (transplant candidate) with risk factors of being exposed to the virus during the evaluation stage prior to donation [9] and a desire of the donor to preserve two kidneys in a reality of medical uncertainty [14]. At the same time, it should be noted that the transplant centers reported that precisely during the period of lock-down many altruistic donors sought to advance the date of the transplant, since in any case they were not working at that time—unlike the related living donors who sought to defer the surgery to help the children at home during this period. Hence importance will be given to understanding the reasons for the decrease in related living donations as well as examining with each altruistic donor the most appropriate time to schedule the transplant date.

#### Informed consent

The gap noted between the number of altruistic donors and related living donors during the epidemic also suggested that families of patients awaiting transplantation were relying more on altruistic donations without possibly first exhausting the option of donation from within the family. In a recent survey of attitudes of living kidney donors (unpublished data, 2018), a significant number of altruistic donors were interested in knowing this information, so that they could donate to those who had no other option. When this question arises during the pre-donation process, altruistic donors are answered in the affirmative or negative, without providing details in the event of a negative response. This reliance on altruistic donation, instead of a donation from a relative, may potentially lead to the abuse of the good intentions of altruistic donors and to a decreased incentive for related living donors. This highlighted an important ethical problem and led to important changes being made. Firstly, the NTC is developing guidelines to inform altruistic donors whether a potential recipient has exhausted the donation options in his/her family, or at the very least to inquire if they wish to know whether all donation options have been exhausted. Secondly, the kidney sub-committee of the NTC has emphasized the importance of nephrologists raising the possibility of related living donation with all their patients attending pre-dialysis clinics. Finally, the National Committee approving all living kidney donations will examine with the potential recipient whether all options for related living donation have been exhausted, and if not—then why not.

## Conclusions

This study of the organ donation program during a crisis brought to light previously unknown and unaccounted for scenarios. This has informed the introduction of policy changes in the NTC including the necessity to mobilize rapidly a “war room”, the use of innovative virtual tools for contact-less communication with donor and recipient families as well as with distant or lock-downed experts, and the importance of close cooperation with hospital authorities in allocating scarce health-care resources. Finally, ethical considerations relating to such crises must be taken into account. This is also seen as an opportunity to improve decision-making and policy implementation and many of these innovations will be adopted for use during “normal’ times.

## Data Availability

The data that support the findings of this study are available from the corresponding author upon reasonable request.
